# Palmitate-induced lipotoxicity alters acetylation of multiple proteins in clonal β cells and human pancreatic islets

**DOI:** 10.1038/s41598-017-13908-w

**Published:** 2017-10-18

**Authors:** Federica Ciregia, Marco Bugliani, Maurizio Ronci, Laura Giusti, Claudia Boldrini, Maria R Mazzoni, Sandra Mossuto, Francesca Grano, Miriam Cnop, Lorella Marselli, Gino Giannaccini, Andrea Urbani, Antonio Lucacchini, Piero Marchetti

**Affiliations:** 1 Department of Pharmacy, University of Pisa; Via Bonanno 6, 56126 Pisa, Italy; 2 Department of Clinical and Experimental Medicine, Islet Cell Laboratory, Via Savi 10, 56126 Pisa, Italy; 3 Proteomics and Metabonomics Unit, IRCCS-Fondazione Santa Lucia; Via del Fosso di Fiorano 64/65, 00143 Rome, Italy; 4 Department of Medical, Oral and Biotechnological Sciences, University G. d’Annunzio of Chieti-Pescara; Via Dei Vestini 31, 66013 Chieti, Italy; 5 ULB Center for Diabetes Research, Université Libre de Bruxelles, Brussels, Belgium; 6 Division of Endocrinology, Erasmus Hospital, Université Libre de Bruxelles, Brussels, Belgium; 7 Istituto di Biochimica e Biochimica Clinica, Università Cattolica; Largo Francesco Vito 1, 00168 Roma, Italy

## Abstract

Type 2 diabetes is characterized by progressive β cell dysfunction, with lipotoxicity playing a possible pathogenetic role. Palmitate is often used to examine the direct effects of lipotoxicity and it may cause mitochondrial alterations by activating protein acetylation. However, it is unknown whether palmitate influences protein acetylation in β cells. We investigated lysine acetylation in mitochondrial proteins from INS-1E β cells (INS-1E) and in proteins from human pancreatic islets (HPI) after 24 h palmitate exposure. First, we confirmed that palmitate damages β cells and demonstrated that chemical inhibition of deacetylation also impairs INS-1E function and survival. Then, by 2-D gel electrophoresis, Western Blot and Liquid Chromatography-Mass Spectrometry we evaluated the effects of palmitate on protein acetylation. In mitochondrial preparations from palmitate-treated INS-1E, 32 acetylated spots were detected, with 13 proteins resulting over-acetylated. In HPI, 136 acetylated proteins were found, of which 11 were over-acetylated upon culture with palmitate. Interestingly, three proteins, glutamate dehydrogenase, mitochondrial superoxide dismutase, and SREBP-1, were over-acetylated in both INS-1E and HPI. Therefore, prolonged exposure to palmitate induces changes in β cell protein lysine acetylation and this modification could play a role in causing β cell damage. Dysregulated acetylation may be a target to counteract palmitate-induced β cell lipotoxicity.

## Introduction

Type 2 diabetes (T2D) is a metabolic disorder characterized by progressive β cell dysfunction in the context of a condition of insulin resistance in insulin target tissues^1,2^. The prevalence of T2D in the world has more than doubled during the past 20 years, partially due to rising obesity rates in both developed and developing countries^3^. Indeed, obesity is considered a major risk factor for the development of T2D also due, at least in part, to its association with higher levels of circulating free fatty acids (FFAs)^4^. In particular, increased concentrations of palmitate, the most abundant saturated FFA in blood, have been related to several deleterious effects on biological systems, collectively termed lipotoxicity^5^.

In pancreatic β cells, prolonged exposure to palmitate causes decreased glucose-stimulated insulin secretion and increased apoptosis^6–12^ possibly mediated by endoplasmic reticulum (ER) stress^13^, increased reactive oxygen species (ROS)^14,15^, dysregulated autophagy^6^ and impairment of mitochondrial functions^15–18^. The coupling of glycolysis to mitochondrial ATP production is essential for proper β cell function and insulin exocytosis^18^ and defects in mitochondrial function impair this metabolic coupling and ultimately promote β cell damage^17,18^. Accordingly, in a previous study we observed several changes in INS-1E mitochondrial proteins after exposure to palmitate showing alterations in pathways involved in ATP production, lipid and aminoacid metabolism, oxidative stress, and apoptosis^19^.

One additional - and so far little explored - possibility linking lipotoxicity to β cell mitochondrial damage is the promotion of post-translational protein modifications by palmitate. Post-translational modifications (PTMs) are a fundamental and highly dynamic machinery for the regulation of cellular biological functions. Among PTMs, proteomic studies have identified protein acetylation as an important modification linked to the metabolic state of the cell^20,21^. Protein acetylation was initially discovered as being an essential regulatory process of chromatin dynamics for histones and in recent studies protein lysine acetylation has emerged as a pivotal determinant in metabolic pathways, especially in mitochondria^22–25^. Lysine acetylation is a reversible PTM which involves the transfer of an acetyl moiety to the ε-amino group of lysine. Its levels change between fasting and feeding^20,24^ reflecting the balance between acetyltransferase and deacetylase activity on target lysine residues^26^. High levels of palmitate are expected to increase acetyl-CoA content and the NADH/NAD^+^ ratio. In mitochondria, the increased acetyl-CoA would promote acetylation while the increased NADH/NAD^+^ ratio would compromise the activity of the primary mitochondrial deacetylase, sirtuin 3 (SIRT3), which uses NAD^+^ as a cofactor^24^.

With this scenario in mind, we investigated lysine acetylation in mitochondrial preparations obtained from INS-1E β cells and in protein extracts from isolated human pancreatic islets after prolonged exposure to palmitate. We used two-dimensional gel electrophoresis (2-DE) and Western Blot (WB) analysis to locate the preferentially acetylated proteins, which were subsequently identified by Liquid Chromatography-Mass Spectrometry (LC-MS). The present work contributes to the continuous progress in defining features of lipotoxicity in pancreatic β cells.

## Results

### Function and survival of INS-1E β cells

First we assessed the effects of prolonged palmitate exposure on glucose-stimulated insulin secretion from INS-1E β cells. As shown in Supplementary Fig. 1, insulin release in response to 2.5 and 16.7 mM glucose was not apparently influenced by culture in palmitate-containing medium for 6 and 14 h. However, after 24 h exposure to the fatty acid, INS-1E β cells showed increased basal secretion and impaired ability to proportionately augment the release of insulin at higher glucose concentration; in addition, compared to control samples, palmitate-treated samples showed reduced cell survival (−32.0 ± 9.4%, p < 0.05). These results confirm what reported in previous studies on β cell deleterious effects of palmitate^27,28^. Then, in another set of experiments, INS-1E β cells were exposed for 24 h to a combination of deacetylase inhibitors (trichostatin A and nicotinamide). As illustrated in Supplementary Fig. 1, cells exposed to the deacetylase inhibitors showed a slightly higher release of insulin at basal (2.5 mM) glucose concentration, and the secretion of the hormone at stimulating glucose level (16.7 mM glucose) was significantly lower compared to that of control cells. Furthermore, in the presence of deacetylase inhibition, cell survival was 33.1 ± 10.8% lower than that of cells cultured in control medium (p < 0.05). When INS-1E β cells were incubated for 24 h with both palmitate and deacetylase inhibitors, we observed a further deterioration of insulin release with respect to palmitate alone, particularly at basal conditions (release at 2.5 mM glucose: 3.98 ± 2.74%; p < 0.05; release at 16.7 mM glucose: 3.44 ± 1.33%, ns), which was accompanied by a modest, additional decrease of β cell survival (− 9.1 ± 1.2%, ns).

These results confirm that 24 h exposure to palmitate alters insulin secretion and survival of INS-1E β cells, and demonstrate that inhibition of deacetylation further impairs the function and survival of palmitate-exposed cells.

### Protein acetylation in mitochondria from INS-1E β cells

**Figure 1:**
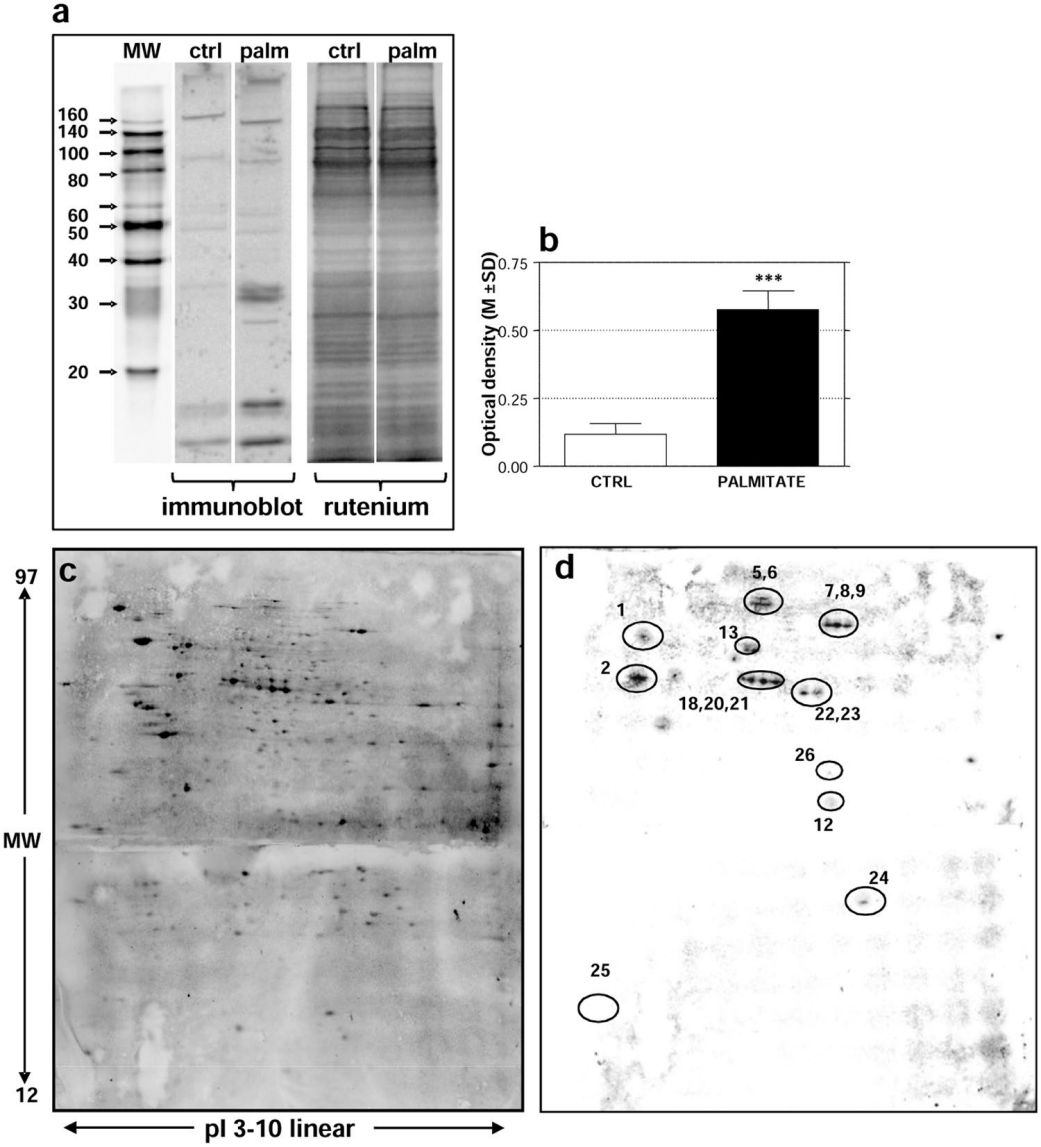
Mitochondria from INS-1E β cells. In order to check the global acetylation level, one-dimensional electrophoresis was performed. Aliquots of mitochondrial samples corresponding to 10 µg of proteins were mixed with Laemmli solution, heated at 100 °C for 5 min, loaded onto 12% polyacrylamide gels and separated by electrophoresis before WB. After protein transfer, membranes were firstly stained with RuBP and subsequently incubated with the specific anti-acetylated lysine antibody. The RuBP-stained images were used for normalization. (**a**) Immunoreactive bands of the mitochondrial enriched fraction from INS-1E β cells detected using an anti acetylated-lysine antibody and the same membrane stained with RuBP. CTRL: control samples: PALM: palmitate-exposed samples. MW: molecular weight standards. (**b**) Histograms of the normalized optical density calculated for antigen-specific bands. The optical density was measured for each band of the whole lane. This density was then normalized by the density of total proteins showed by RuBP staining. Data are presented as mean ± standard deviation of three technical replicates (M ± SD) ***p ≤ 0.001. (**c**) A representative nitrocellulose with 2-DE protein map of mitochondrial enriched fraction of INS-1E β cells. Proteins were detected by RuBP staining. Proteins were firstly separated according to pI on Immobiline Dry-Strips (18 cm, linear gradient pH 3–10) and then according to molecular weight on 12.5% polyacrylamide gels. Then, proteins were transferred onto nitrocellulose membranes. Immediately after WB, membranes were stained with RuBP. (**d**) The same membrane after detection of the immunoreactive spots using the anti-acetylated lysine antibody. Spots of interest are encircled.

Mitochondrial enriched preparations were obtained using differential centrifugations, according to the protocols implemented within the Italian human mitochondrial proteome project (MtHPP) network^29^ and the quality of enrichment was checked as previously described^19^. A mitochondrial protein fraction of approximately 150 µg (starting from 20 million cells) was obtained from each of 7 independent experiments of control and palmitate-treated INS-1E β cells. These mitochondrial preparations were pooled together and analyzed in triplicate. We first verified global acetylation levels by WB analysis. Proteins were separated by 1-D gel electrophoresis and immunoblotted using a specific anti-acetylated lysine antibody. As shown in Fig. 1 panel a, several immunoreactive bands, ranging from 7 to 167 kDa, were detected and an apparent increase of acetylation was observed after palmitate treatment (Fig. 1 panel b).

Then, identification of specific acetylated proteins was performed. To this aim, aliquots of mitochondrial fractions were analyzed by coupling 2-DE and WB (Fig. 1 panel c and d). Immediately after WB, membranes were stained with Ruthenium II tris (bathophenanthroline disulfonate) tetrasodium salt (RuBP) and subsequently incubated with the specific anti-acetylated lysine antibody. This procedure allowed us to exactly match the image of the stained membrane containing all transferred proteins (Fig. 1, panel c) with the image of the same membrane obtained by detecting the immunoreactive spots (Fig. 1, panel d). Each spot intensity was normalized by the total protein intensity as visualized in the image of membrane stained with RuBP. Overall, we detected 32 acetylated spots, of which 17 were over-acetylated in palmitate-treated samples. These differentially acetylated spots were analyzed by tandem mass spectrometry to obtain their identification and collapsed to 13 proteins (Table 1). The molecular weight (MW), isoelectric point (pI), and statistical data of identified proteins are also shown in Table 1. For some proteins (voltage-dependent anion-selective channel protein 2, 60 kDa heat shock protein, succinate dehydrogenase flavoprotein subunit, ATP synthase subunit δ, pyruvate dehydrogenase E1 component subunit α, protein disulfide-isomerase A3) the corresponding acetylated spot was absent in the control samples. This finding does not necessarily indicate the absence or under expression of the protein but only of its acetylated form. Four of these proteins (78 kDa glucose-regulated protein, protein disulfide-isomerase A3, sterol regulatory element-binding protein 1 (SRBP1) and protein disulfide-isomerase A1) belong to the ER, which is partially physically and functionally associated with mitochondria^30–33^.

**Table 1:** Mitochondria from INS-1E β cells.

**n°**	**ID**	**Description**	**Protein**	**Gene**	**a**	**MW**		**pI**		control*	palmitate*	***p*** **-value**
						***obs***	***th***	***obs***	***th***	**M ± SD**	**M ± SD**	
**1**	P06761	78 kDa glucose-regulated protein	GRP78	*Hspa5*	401	74	72	4.8	5.1	1.98 ± 0.4	2.59 ± 0.6	0.05
**2**	P04785	Protein disulfide-isomerase	PDIA1	*P4hb*	625	61	57	4.6	4.8	2.76 ± 0.5	6.57 ± 1.3	0.0098
**5**	P52873	Pyruvate carboxylase	PYC	*Pc*	76	110	130	6.4	6.3	0.70 ± 0.1	4.36 ± 0.8	0.002
**6**	P56720	Sterol regulatory element-binding protein 1	SRBP1	*Srebf1*	45	100	120	6.2	8.4	1.25 ± 0.3	2.11 ± 0.4	0.038
**7**	Q9ER34	Aconitate hydratase	ACON	*Aco2*	74	85	85	7.0	7.9	2.12 ± 0.4	3.21 ± 0.6	0.035
**8**	Q9ER34	Aconitate hydratase	ACON	*Aco2*	95	85	85	7.1	7.9	2.39 ± 0.5	3.42 ± 0.7	n.s.
**9**	Q9ER34	Aconitate hydratase	ACON	*Aco2*	65	85	85	7.2	7.9	2.20 ± 0.4	3.19 ± 0.6	0.045
**12**	P81155	Voltage-dependent anion-selective channel protein 2	VDAC2	*Vdac2*	68	35	32	6.6	7.4	—	0.85 ± 0.2	<0.0001
**13**	Q920L2	Succinate dehydrogenase flavoprotein subunit	DHSA	*Sdha*	50	72	72	6.2	6.7	—	1.07 ± 0.2	<0.0001
**18**	P63039	60 kDa heat shock protein	CH60	*Hspd1*	25	62	61	6	5.9	—	1.44 ± 0.3	<0.0001
**20**	P11598	Protein disulfide-isomerase A3	PDIA3	*Pdia3*	57	62	57	6.2	5.9	—	4.40 ± 0.9	<0.0001
**21**	P11598	Protein disulfide-isomerase A3	PDIA3	*Pdia3*	31	62	57	6.3	5.9	0.52 ± 0.1	4.72 ± 0.9	0.0016
**22**	P10860	Glutamate dehydrogenase 1	DHE3	*Glud1*	112	57	56	7.0	6.7	1.39 ± 0.3	2.07 ± 0.4	0.05
**23**	P10860	Glutamate dehydrogenase 1	DHE3	*Glud1*	163	57	56	7.2	6.7	0.86 ± 0.2	1.75 ± 0.4	0.017
**24**	P07895	Superoxide dismutase	SODM	*Sod2*	139	28	25	7.5	8.4	1.32 ± 0.3	2.12 ± 0.4	0.049
**25**	P35434	ATP synthase subunit delta	ATPD	*Atp5d*	30	19	18	4.4	5.1	—	0.23 ± 0.1	<0.0001
**26**	P26284	Pyruvate dehydrogenase E1 component subunit alpha	ODPA	*Pdha1*	26	45	44	7.2	9.4	—	1.37 ± 0.4	<0.0001

List of the acetylated protein spots identified by MS. ID: SwissProt accession number, a: mass score, MW: molecular weight, pI: isoelectric point (pI). *Mean ± standard deviation (M ± SD) of the % volume of acetylated spots.

Mitochondrial fractions from untreated and palmitate-treated cells were also digested into peptides and lysine-acetylated peptides were immune-captured using the specific anti-acetyl lysine antibody. The lists of proteins inferred from the identification of the acetylated peptides are supplied as Supplementary Tables S1 and S2. Consistently, the number of detected acetylation sites was higher in the mitochondrial fraction of palmitate-treated cells compared to control cells.

### Protein acetylation in human pancreatic islets

**Figure 2:**
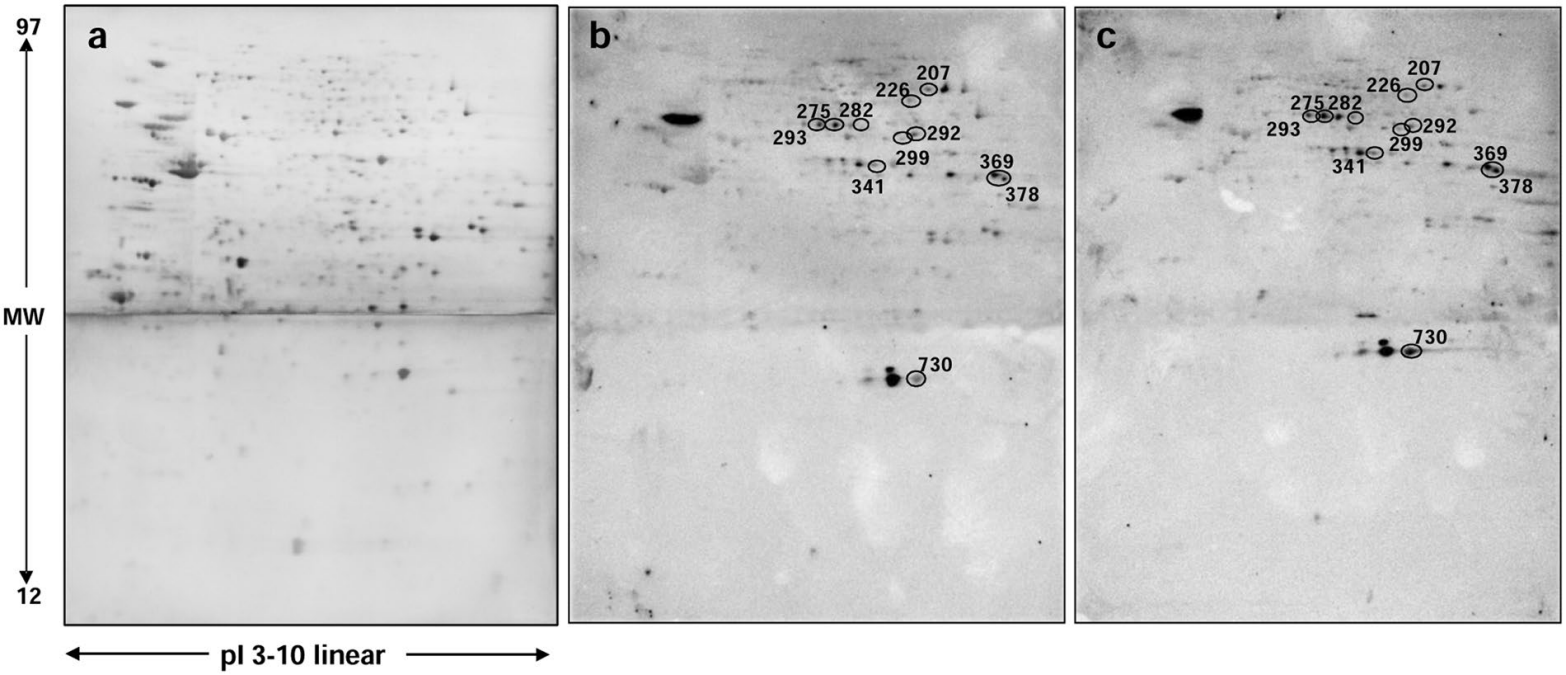
2-DE and WB of human pancreatic islets. 2-DE coupled with WB was employed to detect specific acetylated proteins. Proteins from human islets were firstly separated according to pI on Immobiline Dry-Strips (18 cm, linear gradient pH 3–10) and then according to molecular weight using 12.5% polyacrylamide gels. Subsequently, proteins were transferred onto nitrocellulose membranes. Immediately after WB, membranes were stained with RuBP. Thereafter, membranes were incubated with the anti-acetylated lysine antibody. (**a**) A representative nitrocellulose image with 2-DE protein map of human pancreatic islets (control). Proteins were detected by RuBP staining. (**b**) The same membrane with detection of the immunoreactive spots. (**c**) A representative nitrocellulose membrane with detection of the immunoreactive spots of human pancreatic islets from the same donor as in panel (**a**) but treated with palmitate. Spot of interest are encircled.

**Figure 3:**
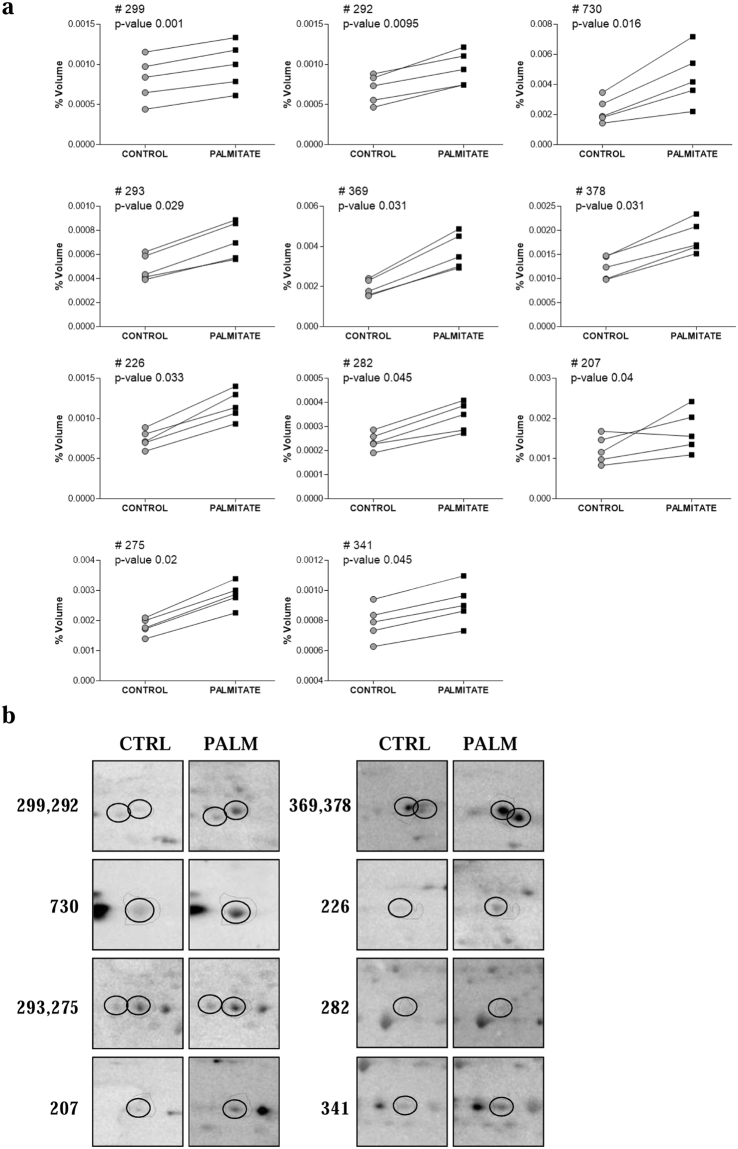
Acetylated proteins in human pancreatic islets. (**a**) The graphical representation of each spot volume (%) obtained by analyzing human pancreatic islets either treated with or without palmitate. Spot number and corresponding identified protein are listed in Table 2. Each pair of connected points represents one experiment using a sample of human pancreatic islets obtained from a single subject. The p-values were determined by paired t test. (**b**) Enlarged images of the acetylated spots detected in human pancreatic islets. CTRL: control samples; PALM: palmitate-exposed samples.

We subsequently analyzed pancreatic islets from 5 non-diabetic human donors. For each experiments, islets were incubated for 24 h with or without palmitate. Approximately 350 µg of protein was extracted from each preparation. Acetylated proteins in human pancreatic islets (Fig. 2) were examined as described above for proteins from INS-1E mitochondrial preparations. We detected 136 acetylated spots and 11 of them were found to be differentially over-acetylated in palmitate-exposed islets (see the histograms in Fig. 3, panel A and Table 2). Interestingly, 3 of these proteins, namely glutamate dehydrogenase (DHE3), superoxide dismutase (SODM) and sterol regulatory element-binding protein 1 (SRBP1), were consistent with those identified in INS-1E β cells.

**Table 2:** Human pancreatic islets.

**n°**	**ID**	**Description**	**Protein**	**Gene**	**a**	**MW**		**pI**		**p-value**
						***obs***	***th***	***obs***	***th***	
299	P00367	Glutamate dehydrogenase 1	DHE3	*GLUD1*	156	61	61	7.2	7.7	0.001
292	P35908	Keratin type II cytoskeletal 2	K22E	*KRT2*	58	65	65	8.2	8.1	0.0095
730	P04179	Superoxide dismutase	SODM	*SOD2*	661	25	25	8.4	8.4	0.016
293	Q71U36	Tubulin alpha-1A chain	TBA1A	*TUBA1A*	75	66	50	6.6	4.9	0.029
369	P04264	Keratin. type II cytoskeletal 1	K2C1	*KRT1*	140	43	66	8.7	8.2	0.031
378	P00966	Argininosuccinate synthase	ASSY	*ASS1*	22	44	47	8.7	8.1	0.031
226	P14866	Heterogeneous nuclear ribonucleoprotein L	HNRPL	*HNRNPL*	108	89	64	8.1	8.5	0.033
282	P11413	Glucose-6-phosphate 1-DH	G6PD	*G6PD*	43	68	59	6.7	6.4	0.045
207	P36956	Sterol regulatory element-binding protein 1	SRBP1	*SREBF1*	57	120	122	8.4	8.4	0.04
275	P55809	Succinyl-CoA:3-ketoacid coenzyme A transferase 1	SCOT1	*OXCT1*	120	68	56	6.5	7.1	0.02
341	Q9NUJ1	Mycophenolic acid acyl-glucuronide esterase	ABHDA	*ABHD10*	34	51	34	6.9	8.8	0.045

List of the acetylated protein spots identified by MS. ID: SwissProt accession number, a: mass score, MW: molecular weight, pI: isoelectric point (pI).

## Discussion

To the best of our knowledge, this is the first study showing that: 1) palmitate exposure induces acetylation of mitochondrial proteins in insulin secreting INS-1E β cells and 2) some of these proteins are also over-acetylated in primary human pancreatic islets. Additional findings of the present report are the confirming evidence that palmitate-treated cells show impaired insulin release capability as well as reduced viability and the novel data indicating that treatment with deacetylase inhibitors also impairs β cell function and survival.

The possibility that palmitate might induce protein acetylation was already suggested in our previous study^19^ where 2-D gel analysis showed the presence of two close spots identified as DHE3 among those found differentially expressed in palmitate treated INS-1E β cells^19^. The present work now demonstrates that 24 h exposure to 0.5 mM palmitate causes over-acetylation of several proteins in INS-1E mitochondrial preparations.

As recently reviewed^34^ protein acetylation in mitochondria typically leads to loss of function, which, in turn, causes alterations of mitochondria integrity. Interestingly, three of the proteins that we identified in INS-1E β cell preparations (DHE3, SODM and the ER protein SRBP1) were also found over-acetylated in palmitate-exposed human pancreatic islets.

All the proteins that we observed to show increased acetylation in the present study are associated with important β cell functions. The mechanisms whereby glucose stimulates insulin secretion require the metabolism of glucose to pyruvate. In pancreatic β cells pyruvate metabolism also depends on the activity of the pyruvate dehydrogenase complex (PDH) and pyruvate carboxylase (PC)^35^. We found that palmitate exposure induced PDH and PC hyper-acetylation. PDH, the rate-limiting enzyme of glucose oxidation, catalyzes the irreversible decarboxylation of pyruvate into acetyl-CoA and its enzymatic activity is under acetylation control^36,37^. SIRT3 increases PDH activity while aberrant PDH hyperacetylation might result in metabolic reprogramming^38^. PC catalyzes the ATP-dependent carboxylation of pyruvate to form oxaloacetate and its activity is decreased in diabetic pancreatic islets compared to non-diabetic samples^39^. It has been previously reported that long-term exposure of rodent β cells to palmitate reduces PC expression and activity^40^. Since we found PC acetylation after cell exposure to palmitate this PTM could be responsible for the described decrease of enzyme activity.

It has been proposed that palmitate hampers mitochondrial function by causing a decrease in cellular ATP production. ATP is needed for the elevation of cytosolic calcium which is required for insulin exocytosis^41^. In our study, acetylated ATP synthase levels were greater in mitochondria from palmitate-treated INS-1E β cells than control cells. These data are consistent with findings showing that acetylation decreases ATP synthase catalytic activity. Indeed, ATP synthase contains multiple SIRT3-dependent reversible acetyl-lysines, which are altered in several conditions of metabolic stress, including exercise, calories restriction, fasting, and high-fat diet^42^. In particular, acetylation is accompanied by a decrease in the activity of this protein^43,44^.

Chronic exposure to FFAs is known to induce β cells apoptosis^8,9,45^. Voltage-dependent anion channels (VDAC) are considered key players in mitochondria-mediated apoptosis, but the molecular mechanisms by which VDAC influences cell death execution are not totally understood^46^. VDAC was shown to be co-translationally or post-translationally modified by phosphorylation or acetylation^21,46,47^ but the relevance of these PTMs in the regulation of VDAC function remains unclear. Interestingly, it was reported that lipid accumulation triggers PTM of VDAC, causing early mitochondrial dysfunction and apoptosis^46,48^. Herein, we detected over-acetylation of VDAC1 by immunoprecipitation (IP) and VDAC2 by WB in mitochondrial preparations from palmitate-treated INS-1E β cells. The molecular consequence of this modification deserves further studies for better understanding the pathophysiological relevance of the various VDAC PTMs especially considering that they can behave as a lipid sensor^46,48^.

A major finding of the present study is that three proteins (DHE3, SODM and SRBP1) were over-acetylated in both INS-1E preparations and human pancreatic islets after exposure to palmitate. DHE3 catalyzes the oxidative deamination of endogenous glutamate, which is present at high concentrations in pancreatic β cells contributing to insulin secretion^49–51^. Post-translational DHE3 acetylation regulates the enzyme activity with progressive loss of activity as the degree of acetylation increases^52,53^. On the other hand, SIRT3 is able to deacetylate and therefore activate DHE3^52,54^. Therefore the impaired DHE3 activity can contribute to the detrimental effect of fatty acids on insulin secretion.

Pancreatic β cells are vulnerable to oxidative stress due to the relatively low expression of antioxidant enzymes^55,56^. Since palmitate can increase the intracellular production of ROS^57^, it can also cause β cell damage by enhancing oxidative stress. In this context, the role of mitochondrial SODM is crucial. This enzyme plays a pivotal role against oxidative stress since mitochondria dissipate over 90% of intracellular oxygen producing a large flux of ROS^58^. The genetic association of *sod2* with T2D has been already reported^59,60^ and the interest in the post-translational regulation of SODM is growing. As a result of palmitate treatment, we observed increased SODM acetylation both in INS-1E β cells mitochondria and human pancreatic islets. Acetylation inhibits SODM catalytic activity while, at the same time, palmitate increases NADH/NAD^+^ ratio impairing SIRT3 activity, which deacetylates and hence activates SODM^58^. Therefore we speculate that the β cell oxidative stress may be also due to the enhanced acetylation by palmitate of key antioxidant enzymes.

In our study we also found over-acetylated proteins of the ER which is strictly associated with the mitochondria and can be present in mitochondrial enriched cellular preparations^30–33^. Among these, we identified the SRBP1, which is a member of the sterol regulatory element binding-proteins (SRBPs), transcriptional factors that control cholesterol and lipid metabolism^61^. SRBP1 mRNA expression is induced in palmitate-treated INS-1E β cells^40^. In our study, we detected increased acetylation levels of SRBP1 in palmitate-treated INS-1E β cells and human pancreatic islets. Moreover acetylation was directly detectable during identification of the corresponding spot by MS (data not shown) in INS-1E β cells and human pancreatic islets. A previous work showed that the activity of SRBPs is regulated by PTMs including acetylation^62^. Of interest, these enzymes are important targets of SIRT1^63^. In particular, acetylation levels were found to be increased in response to feeding and decreased by fasting and therefore it was proposed that deacetylation by SIRT1 inhibits SRBP^63,64^. Indeed, SIRT1 decreases the stability of SRBP through deacetylation, which in turn affects the level of occupancy at the lipogenic target gene promoters^63^. On the other hand, acetylation enhances SRBP activity by increasing its stability and recruitment to its lipogenic target promoters, which favours lipogenesis and contribute to fatty liver disease (hepatosteatosis), a condition associated with obesity and insulin resistance^63^. Therefore, since acetylation of SRBPs is considered a crucial event for the pathogenesis of metabolic disorders^64–66^ its deacetylation through SIRT1 targeting could be useful in the treatment of T2D.

**Figure 4:**
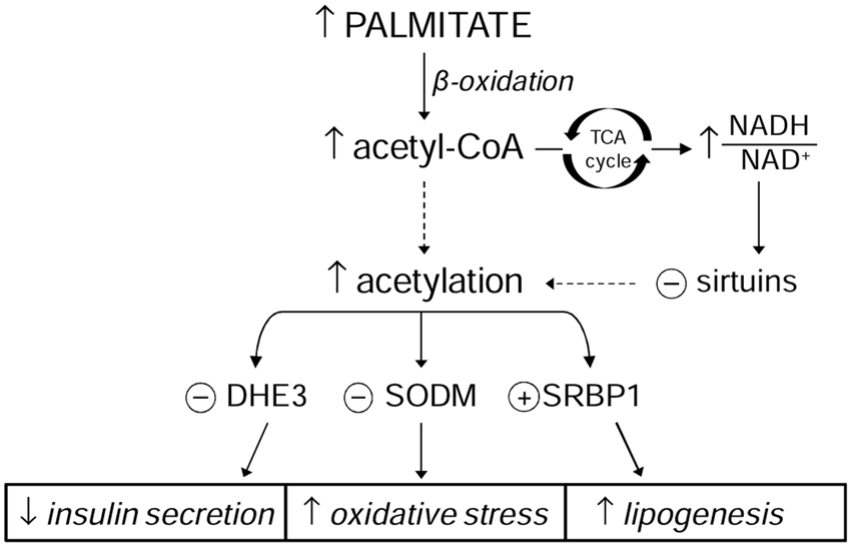
Possible mechanisms linking palmitate exposure, protein (DH3, SODM and SREBP-1) over-acetylation and cellular functions High levels of palmitate are expected to increase both acetyl-CoA content and NADH/NAD^+^ ratio, through increased β-oxidation. The increased acetyl-CoA would promote acetylation, while the increased NADH/NAD^+^ ratio would compromise the activity of SIRTs which use NAD^+^ as a cofactor. The increased acetylation of DHE3 causes a progressive loss of activity impairing insulin secretion. SODM plays a pivotal role against oxidative stress and acetylation inhibits its catalytic activity contributing to oxidative stress. The hyper-acetylation of SRBP1 enhances its activity by increasing its stability and its recruitment to lipogenic promoters, which could promote lipogenesis.

In conclusion, this study demonstrates that prolonged exposure to palmitate induces changes of mitochondrial protein lysine acetylation, which could play a role in β cell dysfunction through several possible mechanisms some of which are summarized in Fig. 4. Therefore, the balance between acetylation and deacetylation may be critical in pancreatic β cell adaptation to lipotoxicity. Whereas additional studies are needed to unveil the fine molecular mechanisms that regulate lysine acetylation/deacetylation pathways the present results provide novel data that could pave the way for future targeted therapeutic strategies modulating acetyl transferase and deacetylase activity.

## Methods

### Cell culture

#### INS-1E β cells

INS-1E β cells (kindly provided by Prof. C. Wollheim Centre Médical Universitaire, Geneva, Switzerland) were handled as previously described^19^. For the purposes of the present study, batches of cells were seeded in 24 well plates at density of 2 × 10^5^ cells/well and cultured up to 24 h in RPMI medium containing 0.5 mM palmitate, 1% fatty acid free-bovine serum albumin (BSA) and 1% FBS. Palmitate was dissolved in ethanol 90% (v/v). The control was represented by cells exposed to the ethanol 90% (v/v).The procedures for the preparation of the palmitate solution have been previously described in detail^67^.

In additional experiments, INS-1E β cells were exposed for 24 h to control or 0.5 mM palmitate containing medium, with or without the addition of deacetylase inhibitors (combined 500 nM trichostatin A and 10 mM nicotinamide)^68^. For insulin release studies, the cells were first exposed to RPMI medium without glucose for 2 h. Then, after a 30 min washing period with a Krebs’ solution containing 2.5 mM glucose, cells were exposed to 2.5 or 16.7 mM glucose for 30 min. At the end, the supernatant was collected and stored at −20 °C until insulin measurement. Total insulin content was extracted by an ethanolic acid solution. Insulin was quantified by the High Range Rat Insulin ELISA kit following the manufacturer’s protocol (Mercodia AB, Uppsala, Sweden). INS-1E β cell viability was monitored using the 3-(4,5-dimethylthiazol-2-yl)-2,5-diphenyltetrazolium bromide-formazan (MTT) assay. Briefly, a solution containing 5 mg/mL of tetrazolium bromide salt (Sigma-Aldrich) dissolved in phosphate buffer saline (PBS) was added to cells exposed to different incubation conditions to obtain a final concentration of 5 μg/mL. Then, plates were kept in an incubator at 37 °C and 5% CO_2_ for 3 h. At the end, supernatants were aspirated and the formed salt dissolved in DMSO. After 20 minutes of shaking, plates were read at 570 nm with baseline at 650 nm. Viability was calculated as percentage of absorbance of treated versus untreated cells.

#### Human pancreatic islets

Islets were isolated by enzymatic digestion and gradient purification^9,69^ from the pancreas of 5 multiorgan donors who gave informed consent (age: 79 ± 6 years; 2 M/3 F; BMI: 25.8 ± 2.5 kg/m^2^), with the approval of the local Ethics Committee (Comitato Etico di Area Vasta Nord Ovest, Azienda Ospedaliera Universitaria Pisana, Pisa) since the glands were not suitable for clinical purposes. The average β cell proportion in islets prepared in our laboratory over the past 10 years is 50 ± 17% (mean ± standard deviation of 163 preparations). In 2 of the 5 islet preparations used in the present study, the percentage of β cells was evaluated: insulin immunofluorescence staining after islet dispersion^70^ identified 40 and 48% β cells. Following isolation, islets were cultured in M199 medium containing 5.5 mM glucose, supplemented with 10% (v/v) serum, 100 U/mL penicillin, 100 μg/mL streptomycin, 50 μg/mL gentamicin, and 750 ng/mL amphotericin B (Sigma-Aldrich, St. Louis, MO). Approximately 1,000 islets were then incubated with or without 0.5 mM palmitate (Sigma-Aldrich) for 24 h, as described above and previously detailed^6,17,69^. All experiments were performed in accordance with relevant guidelines and regulations.

### Mitochondria enrichment

Mitochondrial enriched fractions were obtained from INS-1E β cells using differential centrifugations, according to the protocols implemented within the MtHPP network^29^ and the quality of enrichment was checked as previously described^19^. Briefly, cells (about 20 millions) were collected from the flasks, suspended in the isolation buffer (IB) (20 mM TRIS pH 7.4, 250 mM sucrose, 2 mM EDTA, 50 mM NaF, 2 mM Na_3_VO_4_, 1 μL/10^6^ cells protease inhibitors, 1 µM trichostatin A, 10 mM nicotinamide), transferred into a pre-cooled potter and homogenized. Consecutive centrifugation cycles were used to isolate mitochondria from unbroken cells, nuclei and cytosol. Finally, a volume of IB, depending on the size of the pellet, was added to resuspend the mitochondrial fraction. Protein amounts were determined by the Bio-Rad DC-protein assay (Bio-Rad; Hercules, CA, USA) using BSA as calibration standard. Mass spectrometry analysis of our mitochondrial preparations showed protein yields of 45 ± 7% and 20 ± 5% respect to total proteins, for mitochondrial and ER proteins, respectively. These yields were in accordance to previous results^19,29^. The integrity of mitochondria was assayed by measuring the activity of cytochrome oxidase (Cytochrome Oxidase Activity Colorimetric Assay Kit; BioVision, CA, USA). WB analysis was performed to confirm the enrichment of the mitochondria using a cocktail of 4 different antibodies each targeting a specific cellular marker (1:250 dilution; Abcam, UK) as previously described^19^. Mitochondria preparations which were isolated as described above from seven independent experiments consisting of palmitate-treated and -untreated cells (control) were pooled to perform proteomics and WB analysis.

### Proteins extraction from human pancreatic islets

Isolated islets were collected and washed twice with PBS (37 °C). Cells were suspended in the rehydration solution (7 M urea, 2 M thiourea, 4% CHAPS, 60 mM dithiothreitol (DTT), 0.002% bromophenol blue) added with 50 mM NaF, 2 mM Na_3_VO_4_, 1 μL/10^6^ cells protease inhibitors, 1 µM trichostatin A, 10 mM nicotinamide. After stirring and sonication (4 seconds, 5 times) cells were allowed to rehydrate for 1 h at room temperature (RT) with occasional stirring. Thereafter, the solution was centrifuged at 17,000 *g* for 5 min at RT. Protein concentration of the resulting supernatant was determined using the Bio-Rad RC/DC-protein assay (Bio-Rad). BSA was used as a standard. The global protein content was analyzed with focus on mitochondrial proteins.

### Electrophoresis and Western Blot

#### Sodium Dodecyl Sulphate-Polyacrylamide Gel Electrophoresis (SDS-PAGE)

In order to check the global acetylation level, 1-D gel electrophoresis was performed. Aliquots of mitochondrial samples corresponding to 10 µg of proteins were mixed with Laemmli solution and heated at 100 °C for 5 min, loaded onto 12% polyacrylamide gels and separated by electrophoresis (Mini-PROTEAN^®^ Tetra Cell, Bio-Rad) before WB.

#### Two-dimensional electrophoresis

2-DE coupled with WB was employed to detect specific acetylated proteins. 2-DE was carried out as previously described^71^. 2-DE was performed on human pancreatic islets and mitochondria from INS-1E β cells. Briefly, 150 µg of proteins were filled up to 450 μL in rehydration solution. Immobiline Dry-Strips (GE Health Care Europe; Uppsala, Sweden), 18 cm, linear gradient pH 3–10 were rehydrated overnight in the sample and then transferred to the Ettan IPGphor Cup Loading Manifold (GE Healthcare) for isoelectrofocusing (IEF). The second dimension (SDS-PAGE) was carried out by transferring the proteins to 12.5% polyacrylamide gels.

#### Western Blot (WB)

Proteins separated by 1-D and 2-DE gel electrophoresis were transferred onto nitrocellulose membranes (0.2 mm). Immediately after WB, the membranes were fixed in 7% acetic acid (v/v) and 10% methanol (v/v) for 15 min, subsequently rinsed with H_2_O, and stained with 1 mM RuBP (SunaTech Inc.; Suzhou, P. R. China) in 1% phosphoric acid and 30% ethanol for 15 min. Membranes were then rinsed with H_2_O prior to the acquisition of the total proteins by “ImageQuant LAS4010” (GE HealthCare). After acquisition, membranes were blocked with 3% low fat dried milk, 0.2% (v/v) Tween 20 in PBS for 1 h at room temperature. Subsequently, membranes were incubated 2 h at room temperature with the primary antibody for acetylated-lysine (mouse monoclonal; 1:1,000 dilution, Cell Signaling Technology Inc., MA, USA). HRP-conjugated goat antimouse (1:10,000 dilution; PerkinElmer, MA, USA) was used as a secondary antibody. Immunoblots were developed using the ECL detection system (PerkinElmer, MA, USA). The chemiluminescent images were acquired by “ImageQuant LAS4010”.

#### Preparative gels

For the preparative gels 200 µg of proteins were filled up to 450 μl in rehydration solution and 2-DE was carried out as described above. At the end of the second dimension gels were stained with 1 mM RuBP in 1% phosphoric acid and 30% ethanol O/N. 2-DE SDS-PAGE standards (17.5–76 kDa, 4.5–8.5 pH; from BioRad) were used for calibration. The software Image-Master 2D Platinum 6.01 (GE Health Care) was used to match the image of the membrane stained with RuBP with the image of the membrane with acetylated spots and the image of the preparative gel. The protein spots of interest were cut out from the gel and identified by LC-MS analysis.

### Spot digestion and protein identification

The gel pieces were trypsin digested and analyzed by LC-MS as previously described^19^ using a short chromatographic gradient and in autoMS mode. DataAnalysis v. 4.2 was used to process the raw data and generate the peak list to be submitted to the database search through BioTools 3.2 exploiting the free version of MASCOT search engine against Uniprot/Swiss-Prot non-redundant database version 2014-11. *Rattus norvegicus* or *Homo sapiens* taxonomy were specified for database searching. Acetylation of lysines was specifically selected as possible modification together with the usual oxidation of methionines.

### Immunoprecipitation

IP was performed on 400 µg of mitochondria obtained from INS-1E β cells treated with and without palmitate. Mitochondrial preparations were resuspended in 1% Octyl β-D-glucopyranoside in 100 mM NH_4_HCO_3_, and vortexed. Then 10 M urea in 100 mM NH_4_HCO_3_ was added and vortexed until the lysate became clear. Protein disulfide bonds were reduced by 1.8 M DTT in NH_4_HCO_3_ (40 min, 30 °C), and alkylated with 625 mM IAA in NH_4_HCO_3_ (30 min, RT, in the dark), additional 1.8 M DTT in NH_4_HCO_3_ was added and kept 15 min at RT. The solution was then incubated at 37 °C overnight with trypsin porcine (1:40 w/w; Promega). The reaction was quenched by adding 10% formic acid (FA). The resulting peptides were purified using reversed-phase purification cartridges (Oasis^®^; Waters, MA, USA). Purified peptides were eluted using a solution with 80% ACN and 0.1% FA. The elutes were dried in SpeedVac Concentrator (Thermo Scientific; Bremen, Germany). Then, the dried elutes were redissolved in IP buffer (50 mM Tris, pH 8.0, 1 mM EDTA, 100 mM NaCl) and incubated with beads (Protein A/G PLUS-Agarose; Santa Cruz Biotechnology Inc., Dallas, TX, USA) and anti-acetylated lysine antibody (1:12.5 w/w; mouse monoclonal; Cell Signaling Technology Inc.) for 18 hrs at 4 °C on a rotation wheel. The immunoprecipitated peptides were washed 3 times with IP buffer, eluted using 1% TFA, 40% acetonitrile in H_2_O and dried in SpeedVac Concentrator. After re-solubilisation in 0.1% FA, the enriched acetylated peptides were analyzed by LC-MS as already reported^19^. Raw data were processed with PEAKS 7.5 to provide protein IDs and PTMs mapping.

### Data analysis

Images from WB of 1-D gel electrophoresis were analyzed using the Image Quant-TL (GE Health Care). The antigen-specific bands and the total proteins after RuBP staining were quantified. The optical density was measured for all the immunoreactive bands of the whole lane. In order to normalize the volume of each band, the ratio of optical density of the antigen-specific bands with those of total proteins, obtained from RuBP staining, was calculated. The results were expressed as a ratio of optical density. The significance of the differences of global acetylation was calculated by unpaired Student’s t-test making a comparison between the two classes.

The analysis of 2-DE images was performed using the Same Spot (v4.1, TotalLab; Newcastle Upon Tyne, UK) software. The volume of each spot obtained after the detection of immunocomplexes was normalized by the total protein content obtained with RuBP staining of membranes. A comparison between cells treated with and without palmitate was performed. The significance of the differences of normalized volume for each acetylated spot was calculated by paired Student’s t-test. Therefore the protein spots of interest were selected and preparative gels for the identification were prepared.

### Data Availability

All data generated or analyzed during this study are included in this published article (and its Supplementary Information files). The data not shown are available from the corresponding author.

## Electronic supplementary material

Supplementary information

## References

[1] Halban PA (2014). β-cell failure in type 2 diabetes: postulated mechanisms and prospects for prevention and treatment. Diabetes Care.

[2] Marchetti, P. & Ferrannini, E. β-Cell mass and function in human type 2 diabetes. In *International Textbook of Diabetes* Mellitus (ed. De Fronzo, R. A., Ferrannini, E., Zimmet, P., Alberti, G.) 354–370 (John Wiley & Sons, Ltd, 2015).

[3] Zimmet PZ, Magliano DJ, Herman WH, Shaw JE (2014). Diabetes: a 21st century challenge. Lancet Diabetes Endocrinol..

[4] Karpe F, Dickmann JR, Frayn KN (2011). Fatty acids, obesity, and insulin resistance: time for a reevaluation. Diabetes.

[5] Ertunc ME, Hotamisligil GS (2016). Lipid signaling and lipotoxicity in metaflammation: indications for metabolic disease pathogenesis and treatment. J. Lipid. Res..

[6] Cnop M (2014). RNA sequencing identifies dysregulation of the human pancreatic islet transcriptome by the saturated fatty acid palmitate. Diabetes.

[7] Grill V, Qvigstad E (2000). Fatty acids and insulin secretion. Br. J. Nutr..

[8] El-Assaad W (2003). Saturated fatty acids synergize with elevated glucose to cause pancreatic β-cell death. Endocrinology.

[9] Lupi R (2002). Prolonged exposure to free fatty acids has cytostatic and pro-apoptotic effects on human pancreatic islets: evidence that β-cell death is caspase mediated, partially dependent on ceramide pathway, and Bcl-2 regulated. Diabetes.

[10] Cnop M (2005). Mechanisms of pancreatic β-cell death in type 1 and type 2 diabetes: many differences, few similarities. Diabetes..

[11] Shimabukuro M, Zhou YT, Levi M, Unger RH (1998). Fatty acid-induced β cell apoptosis: a link between obesity and diabetes. Proc Natl Acad Sci USA.

[12] Lee SH (2017). High-throughput screening and bioinformatic analysis to ascertain compounds that prevent saturated fatty acid-induced β-cell apoptosis. Biochem Pharmacol..

[13] Cunha DA (2008). Initiation and execution of lipotoxic ER stress in pancreatic β-cells. J. Cell. Sci..

[14] Natalicchio A (2015). Thep66(Shc) redox adaptor protein is induced by saturated fatty acids and mediates lipotoxicity-induced apoptosis in pancreatic β cells. Diabetologia.

[15] Carlsson C, Borg LA, Welsh N (1999). Sodium palmitate induces partial mitochondrial uncoupling and reactive oxygen species in rat pancreatic islets *in vitro*. Endocrinology.

[16] Lowell BB, Shulman GI (2005). Mitochondrial dysfunction and type 2 diabetes. Science.

[17] Cunha DA (2012). Death protein 5 and p53-upregulated modulator of apoptosis mediate the endoplasmic reticulum stress-mitochondrial dialog triggering lipotoxic rodent and human β-cell apoptosis. Diabetes.

[18] Barlow J, Jensen VH, Jastroch M, Affourtit C (2016). Palmitate-induced impairment of glucose-stimulated insulin secretion precedes mitochondrial dysfunction in mouse pancreatic islets. Biochem. J..

[19] Ciregia F (2015). Glucagon-like peptide 1 protects INS-1E mitochondria against palmitate-mediated β-cell dysfunction: a proteomic study. Mol. Biosyst..

[20] Kim SC (2006). Substrate and functional diversity of lysine acetylation revealed by a proteomics survey. Mol. Cell..

[21] Choudhary C (2009). Lysine acetylation targets protein complexes and co-regulates major cellular functions. Science.

[22] Pougovkina O (2014). Mitochondrial protein acetylation is driven by acetyl-CoA from fatty acid oxidation. Hum. Mol. Genet..

[23] Zhao S (2010). Regulation of cellular metabolism by protein lysine acetylation. Science.

[24] Vadvalkar SS (2013). Metabolic inflexibility and protein lysine acetylation in heart mitochondria of a chronic model of type 1 diabetes. Biochem. J..

[25] Hirschey MD (2011). SIRT3 deficiency and mitochondrial protein hyperacetylation accelerate the development of the metabolic syndrome. Mol. Cell..

[26] Philp A, Rowland T, Perez-Schindler J, Schenk S (2014). Understanding the acetylome: translating targeted proteomics into meaningful physiology. Am. J. Physiol. Cell. Physiol..

[27] Natalicchio A (2016). Long-Term Exposure of Pancreatic β-Cells to Palmitate Results in SREBP-1C-Dependent Decreases in GLP-1 Receptor Signaling via CREB and AKT and Insulin Secretory Response. Endocrinology..

[28] Sol EM, Sargsyan E, Akusjärvi G, Bergsten P (2008). Glucolipotoxicity in INS-1E cells is counteracted by carnitine palmitoyltransferase 1 over-expression. Biochem Biophys Res Commun..

[29] Alberio, T. *et al*. Towards the standardization of mitochondrial proteomics: the Italian mt-HPP initiative. *J Prot Re*s doi: 10.1021/acs.jproteome.7b00350. (2017). 10.1021/acs.jproteome.7b00350 28828861

[30] Marchi S, Patergnani S, Pinton P (2014). The endoplasmic reticulum–mitochondria connection: One touch, multiple functions. Biochim Biophys Acta..

[31] Csordás G (2006). Structural and functional features and significance of the physical linkage between ER and mitochondria. J Cell Biol..

[32] Fernández-Vizarra E (2010). Isolation of mitochondria for biogenetical studies: An update. Mitochondrion..

[33] Thoudam T, Jeon JH, Ha CM, Lee IK (2016). Role of Mitochondria-Associated Endoplasmic Reticulum Membrane in Inflammation-Mediated Metabolic Diseases. Mediators. Inflamm..

[34] Baeza J, Smallegan MJ, Denu JM (2016). Mechanisms and Dynamics of Protein Acetylation in Mitochondria. Trends Biochem Sci..

[35] Sugden MC, Holness MJ (2011). The pyruvate carboxylase-pyruvate dehydrogenase axis in islet pyruvate metabolism: Going round in circles?. Islets.

[36] Rardin MJ (2013). Label-free quantitative proteomics of the lysine acetylome in mitochondria identifies substrates of SIRT3 in metabolic pathways. Proc. Natl. Acad. Sci. USA.

[37] Mori J (2013). Ang II causes insulin resistance and induces cardiac metabolic switch and inefficiency: a critical role of PDK4. Am. J. Physiol. Heart Circ. Physiol..

[38] Ozden O (2014). SIRT3 deacetylates and increases pyruvate dehydrogenase activity in cancer cells. Free Radic. Biol. Med..

[39] MacDonald MJ (2009). Decreased levels of metabolic enzymes in pancreatic islets of patients with type 2 diabetes. Diabetologia.

[40] Cunha DA (2014). JunB protects β-cells from lipotoxicity via the XBP1-AKT pathway. Cell Death Differ.

[41] Supale S, Li N, Brun T, Maechler P (2012). Mitochondrial dysfunction in pancreatic β cells. Trends Endocrinol. Metab..

[42] Vassilopoulos A (2014). SIRT3 deacetylates ATP synthase F1 complex proteins in response to nutrient- and exercise-induced stress. Antioxid. Redox Signal..

[43] Kerner J (2015). Acetyl-L-carnitine increases mitochondrial protein acetylation in the aged rat heart. Mech. Ageing Dev..

[44] Hosp F (2016). Lysine acetylation in mitochondria: From inventory to function. Mitochondrion.

[45] Piro S (2002). Chronic exposure to free fatty acids or high glucose induces apoptosis in rat pancreatic islets: possible role of oxidative stress. Metabolism.

[46] Martel C, Wang Z, Brenner C (2014). VDAC phosphorylation, a lipid sensor influencing the cell fate. Mitochondrion.

[47] Sol EM (2012). Proteomic investigations of lysine acetylation identify diverse substrates of mitochondrial deacetylase sirt3. PLoS One.

[48] Martel (2013). Glycogen synthase kinase 3-mediated voltage-dependent anion channel phosphorylation controls outer mitochondrial membrane permeability during lipid accumulation. Hepatology.

[49] Maechler P, Wollheim CB (2001). Mitochondrial function in normal and diabetic β -cells. Nature..

[50] Li M, Li C, Allen A, Stanley CA, Smith TJ (2014). Glutamate dehydrogenase: structure, allosteric regulation, and role in insulin homeostasis. Neurochem. Res..

[51] Fahien LA, Macdonald MJ (2011). The complex mechanism of glutamate dehydrogenase in insulin secretion. Diabetes.

[52] Pacella-Ince L, Zander-Fox DL, Lan M (2014). Mitochondrial SIRT3 and its target glutamate dehydrogenase are altered in follicular cells of women with reduced ovarian reserve or advanced maternal age. Hum. Reprod..

[53] Colman RF, Frieden C (1966). On the role of amino groups in the structure and function of glutamate dehydrogenase. II. Effect of acetylation on molecular properties. J. Biol. Chem..

[54] Schlicker C (2008). Substrates and regulation mechanisms for the human mitochondrial sirtuins Sirt3 and Sirt5. J. Mol. Biol..

[55] Lin N, Chen H, Zhang H, Wan X, Su Q (2012). Mitochondrial reactive oxygen species (ROS) inhibition ameliorates palmitate-induced INS-1 β cell death. Endocrine.

[56] Lenzen S, Drinkgern J, Tiedge M (1996). Low antioxidant enzyme gene expression in pancreatic islets compared with various other mouse tissues. Free Radic. Biol. Med..

[57] Nakamura S (2009). Palmitate induces insulin resistance in H4IIEC3 hepatocytes through reactive oxygen species produced by mitochondria. J. Biol. Chem..

[58] Chen Y (2011). Tumour suppressor SIRT3 deacetylates and activates manganese superoxide dismutase to scavenge ROS. EMBO Rep..

[59] Nakanishi S (2008). Manganese superoxide dismutase Ala16Val polymorphism is associated with the development of type 2 diabetes in Japanese-Americans. Diabetes Res. Clin. Pract..

[60] Miao L, St. Clair DK (2009). Regulation of superoxide dismutase genes: implications in disease. Free Radic. Biol. Med..

[61] Horton JD, Bashmakov Y, Shimomura I, Shimano H (1998). Regulation of sterol regulatory element binding proteins in livers of fasted and refed mice. Proc. Natl. Acad. Sci. USA..

[62] Giandomenico V, Simonsson M, Grönroos E, Ericsson J (2003). Coactivator-dependent acetylation stabilizes members of the SREBP family of transcription factors. Mol. Cell. Biol..

[63] Ponugoti B (2010). SIRT1 deacetylates and inhibits SREBP-1C activity in regulation of hepatic lipid metabolism. J. Biol. Chem..

[64] Rodgers JT, Puigserver P (2007). Fasting-dependent glucose and lipid metabolic response through hepatic sirtuin 1. Proc. Natl. Acad. Sci. USA.

[65] Osborne TF (2000). Sterol regulatory element-binding proteins (SREBPs): key regulators of nutritional homeostasis and insulin action. J. Biol. Chem.

[66] Foufelle F, Ferré P (2002). New perspectives in the regulation of hepatic glycolytic and lipogenic genes by insulin and glucose: a role for the transcription factor sterol regulatory element binding protein-1c. Biochem. J..

[67] Oliveira AF (2015). *In vitro* use of free fatty acids bound to albumin: A comparison of protocols. Biotechniques..

[68] Palmieri EM (2015). Acetylation of human mitochondrial citrate carrier modulates mitochondrial citrate/malate exchange activity to sustain NADPH production during macrophage activation. Biochim Biophys Acta..

[69] Bugliani M (2009). The direct effects of tacrolimus and cyclosporin A on isolated human islets: A functional, survival and gene expression study. Islets.

[70] Cunha DA (2017). Pancreatic β-cell protection from inflammatory stress by the endoplasmic reticulum proteins thrombospondin 1 and mesencephalic astrocyte-derived neutrotrophic factor (MANF). J Biol Chem. pii: jbc..

[71] Ciregia F (2013). A multidisciplinary approach to study a couple of monozygotic twins discordant for the chronic fatigue syndrome: a focus on potential salivary biomarkers. J. Transl. Med..

