# Supplementary material for: Palmitate-induced lipotoxicity alters acetylation of multiple proteins in clonal β cells and human pancreatic islets
Source: Sci Rep. 2017 Oct 18;7:13445. doi: 10.1038/s41598-017-13908-w (PMC5647430; doi:10.1038/s41598-017-13908-w)
Supplement: Supplementary file 1 — Supplementary information [file 41598_2017_13908_MOESM1_ESM.pdf]

Palmitate-induced lipotoxicity alters acetylation of multiple proteins in clonal β cells and human pancreatic islets.

Federica Ciregia, Marco Bugliani, Maurizio Ronci, Laura Giusti, Claudia Boldrini, Maria R Mazzoni, Sandra Mossuto, Francesca Grano, Miriam Cnop, Lorella Marselli, Gino Giannaccini, Andrea Urbani, Antonio Lucacchini, Piero Marchetti.

Supplementary Table S1. Mitochondria from INS-1E cells. List of proteins which were identified by immune-captured peptides: control.

prot_accession	prot_description	prot_score	prot_mass	pept_variable modifications
RS15A_RAT	40S ribosomal protein S15a	18	14944	
CH60_RAT	60 kDa heat shock protein	19	61088	
RLA2_RAT	60S acidic ribosomal protein P2	20	11685	
ADT1_RAT	ADP/ATP translocase 1	31	33196	Acetyl (N-term)
ATPA_RAT	ATP synthase subunit alpha	33	59831	
ATPB_RAT	ATP synthase subunit beta	25	56318	
H31_RAT	Histone H3.1	23	15509	2 Acetyl (K)
H4_RAT	Histone H4	46	11360	2 Acetyl (K)
INS2_RAT	Insulin-2	25	12673	
MYH9_RAT	Myosin-9	38	227566	Acetyl (K)
NDUAA_RAT	NADH dehydrogenase [ubiquinone] 1 alpha subcomplex subunit 10	20	40753	
PDIA3_RAT	Protein disulfide-isomerase A3	43	57044	
TBA1B_RAT	Tubulin alpha-1B chain	27	50804	
TBB2A_RAT	Tubulin beta-2A chain	49	50274	
UGGG1_RAT	UDP-glucose:glycoprotein	25	177061	

Supplementary Table S2. **Mitochondria from INS-1E cells.** List of proteins which were identified by immune-captured peptides: palmitate.

prot_accession	prot_description	prot_score	prot_mass	pept_variable modifications
RM22_RAT	39S ribosomal protein L22	18	24252	2 Acetyl (K)
4F2_RAT	4F2 cell-surface antigen heavy chain	27	58150	3 Acetyl (K)
CH60_RAT	60 kDa heat shock protein	82	61088	
GRP78_RAT	78 kDa glucose-regulated protein	127	72473	
ACTA_RAT	Actin, aortic smooth muscle	217	42381	
ACTG_RAT	Actin, cytoplasmic 2	267	42108	
ADT1_RAT	ADP/ATP translocase 1	145	33196	Acetyl (K)
ADT2_RAT	ADP/ATP translocase 2	141	33108	
ATPA_RAT	ATP synthase subunit alpha	461	59831	
ATPB_RAT	ATP synthase subunit beta	361	56318	
ATP5I_RAT	ATP synthase subunit e	33	8249	
CISY_RAT	Citrate synthase	37	52176	
CLH1_RAT	Clathrin heavy chain 1	34	193187	
COX5A_RAT	Cytochrome c oxidase subunit 5A	25	16347	
EF1A1_RAT	Elongation factor 1-alpha 1	60	50424	
ENPL_RAT	Endoplasmin	125	92998	
G3P_RAT	Glyceraldehyde-3-phosphate dehydrogenase	28	36090	2 Acetyl (K)
GNAS1_RAT	Guanine nucleotide-binding protein G(s) subunit alpha isoforms XLas	45	123836	
HS71L_RAT	Heat shock 70 kDa protein 1-like	31	70904	
H2A2A_RAT	Histone H2A type 2-A	77	14087	
H2B1_RAT	Histone H2B type 1	35	13982	
HCDH_RAT	Hydroxyacyl-coenzyme A dehydrogenase	51	34540	
INS2_RAT	Insulin-2	17	12673	
LPPRC_RAT	Leucine-rich PPR motif-containing protein	65	157808	
LRC4B_RAT	Leucine-rich repeat-containing protein 4B	16	76983	
MDHM_RAT	Malate dehydrogenase	71	36117	
M2OM_RAT	Mitochondrial 2-oxoglutarate/malate carrier protein	28	34393	Acetyl (K)
MYH9_RAT	Myosin-9	34	227566	3 Acetyl (K)
NDUS1_RAT	NADH-ubiquinone oxidoreductase 75 kDa subunit	20	80331	
PPID_RAT	Peptidyl-prolyl cis-trans isomerase D	17	41139	
PHB_RAT	Prohibitin	28	29859	
PDIA3_RAT	Protein disulfide-isomerase A3	40	57044	
S61A1_RAT	Protein transport protein Sec61 subunit alpha isoform 1	27	52687	
RAB8A_RAT	Ras-related protein Rab-8A	38	23824	Acetyl (K)
AT2A1_RAT	Sarcoplasmic/endoplasmic reticulum calcium ATPase 1	20	110707	
AT2A2_RAT	Sarcoplasmic/endoplasmic reticulum calcium ATPase 2	27	116347	

AT2A3_RAT	Sarcoplasmic/endoplasmic reticulum calcium ATPase 3	22	117749	
SRBP1_RAT	Sterol regulatory element-binding protein 1	24	121472	Acetyl (K)
GRP75_RAT	Stress-70 protein	68	74097	
TMM97_RAT	Transmembrane protein 97	21	21103	
ECHA_RAT	Trifunctional enzyme subunit alpha	18	83297	
NSF_RAT	Vesicle-fusing ATPase	16	83170	
VDAC1_RAT	Voltage-dependent anion-selective channel protein 1	64	30851	
VPP1_RAT	V-type proton ATPase 116 kDa subunit a isoform 1	18	97063	2 Acetyl (K)

Supplementary Figure 1. Insulin release from from INS1-E β.

Insulin release (expressed as % of insulin content) from INS1-E β cells exposed for 6h (panel a), 14h (panel b) or 24h (panel c) to 0.5 mM palmitate; the effects of deacetylase inhibition are shown in panel d. Data are presented as mean ± SD. *p<0.05 vs LG; #p<0.05 vs LG Ctrl; §p<0.05 vs HG Ctrl.

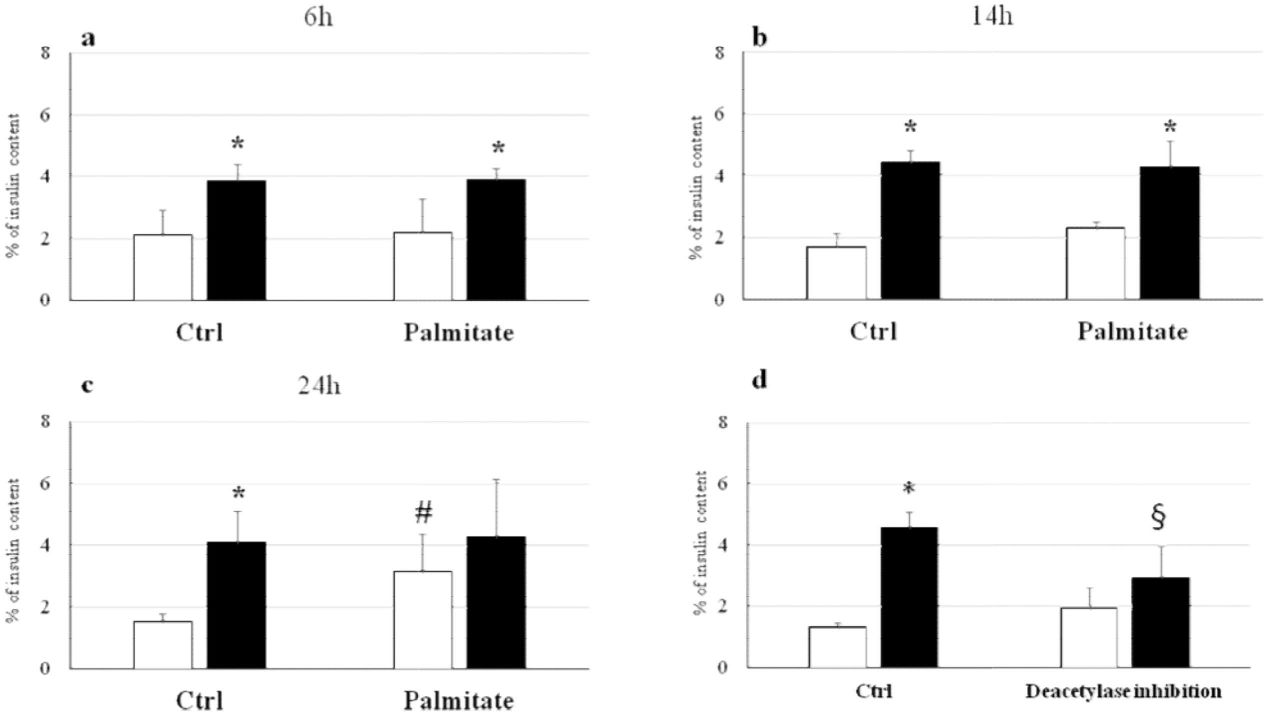

□ 2.5 mM glucose (LG)

■ 16.7 mM glucose (HG)
